# Intersection of Cardiology and Oncology Clinical Practices

**DOI:** 10.3389/fonc.2014.00259

**Published:** 2014-09-24

**Authors:** Farouk Mookadam, Ashwini Sharma, Howard R. Lee, Donald W. Northfelt

**Affiliations:** ^1^Division of Cardiovascular Diseases, Mayo Clinic, Scottsdale, AZ, USA; ^2^Division of Hematology/Oncology, Mayo Clinic, Scottsdale, AZ, USA

**Keywords:** strain imaging, cardiomyopathies, chemotherapy side effects, oncology, cardiovascular diseases

## Introduction

Globally, cancer is diagnosed in approximately 13 million people each year. Approximately 1.6 million cancer patients are seen by cancer clinics across the United States (US) at this time. Over the next two decades, cancer incidence is estimated to increase by approximately 45% to 2.3 million (1). In the US, the 5-year relative survival rate of patients diagnosed with cancer in 1975–1977 was 50%, improving to 68% in the period 1999–2005. Novel targeted chemotherapeutic agents and improved diagnostic techniques are responsible for this increased survival. However, with the improvement in life expectancy, the adverse effects of chemotherapeutic agents, especially cardiotoxicity, is an emerging health problem. Cardiovascular toxicity on its own has a detrimental effect on both the quantity and quality of life independent of the oncological prognosis.

Currently, more than two million women with breast cancer are at risk of anthracycline cardiotoxicity in the US (2). Human epidermal growth factor receptor II (HER2) positive disease comprises approximately 25% of all breast cancer patients and is associated with more aggressive disease activity and worse prognosis. Trastuzumab, a humanized monoclonal antibody used for patients with HER2 positive breast cancer in conjugation with chemotherapy, can provide longer survival and 20% reduction in risk of death (3). Cardiotoxicity becomes an important health issue because up to 27% of women with breast cancer receiving anthracyclines, cyclophosphamide, and trastuzumab showed cardiac dysfunction (3). Breast cancer mortality is reduced by approximately one-third, but the risk of heart toxicity is five times more likely for women receiving trastuzumab than women receiving standard therapy alone (4). Patients showing signs of cardiotoxicity often require a dose reduction, a change in the schedule dosing or even cessation of treatment with attendant consequences. Many patients with an asymptomatic decrease in left ventricular ejection fraction (LVEF) are receiving neither the American College of Cardiology/American Heart Association Class I-indicated treatments nor cardiovascular specialty consultation (5).

Concern for cardiotoxicity is not restricted to breast cancer survivors. Based on National Cancer Institute (NCI) data, the number of new renal cancer patients in 2013 is expected to be 65,000. In Europe, the incidence of renal cell carcinoma (RCC) has doubled in the last three decades (6). Improved treatment strategies have increased the 5-year survival of patients with RCC from 50% in 1975–1977 to 72% in 2002–2008. Within the last decade, the US Food and Drug Administration (FDA) has approved six drugs for the treatment of RCC including multitargeted tyrosine kinase inhibitors (TKIs); antibodies to vascular endothelial growth factor (VEGF); and mammalian target of rapamycin (mTOR) inhibitors. Sunitinib, a novel multitargeted TKI, has proven efficacy in advanced metastatic RCC demonstrating an increased median progression free survival of 8.3 months in these patients (7). In a study by Hall et al. (8), five of the approved targeted therapy drugs (sorafenib, pazopanib, bevacizumab, everolimus, and temsirolimus) have cardiotoxic side effects. In this 159-patient study, 73% of patients experienced some form of cardiotoxicity ranging from hypertension to severe heart failure (8). In a cohort of patients with renal and non-renal carcinoma, sunitinib was found to be associated with a 3.3-fold higher risk of heart failure (9). Other targeted agents such as imatinib mesylate, Dasatinib, Nilotinib, and Sorafenib are prescribed for treatment of various hematological malignancies, hepatocellular carcinoma (HCC), gastrointestinal stromal tumor (GIST), and myeloproliferative/myelodysplastic diseases and have been shown to be strongly associated to cardiotoxicity (10–13). Imatinib has been shown to be associated with decline in LVEF, especially in patients with other comorbidities including coronary artery disease, diabetes, and hypertension (10).

## Current Practice for Detecting Chemotherapy-Induced Cardiotoxicity

The overlap of symptoms between diagnosis of cancer, symptoms of cardiac dysfunction, and the wide spectrum of cardiac injury caused by chemotherapy makes the diagnosis of cardiotoxicity a challenge. These side effects can be categorized as: (a) direct cytotoxic effects of chemotherapy resulting in systolic dysfunction; (b) cardiac ischemia; (c) cardiac arrhythmia; (d) pericarditis; (e) or chemotherapy-induced repolarization abnormalities. Early diagnosis of these abnormalities requires routine baseline and post-chemotherapy monitoring of patients’ cardiac status using symptoms, vital signs, and simple ancillary tests such as an electrocardiogram (ECG), echocardiogram, serum troponin levels, serum brain natriuretic peptide (BNP) where applicable and, less frequently, radionuclide angiocardiography.

The most common practice in the evaluation of cardiac function for patients on chemotherapy is ejection fraction assessment by echocardiography. Cardiotoxicity is most commonly defined as a reduction of the LVEF of >5% to that of a <55% with symptoms of heart failure or an asymptomatic reduction of the LVEF of >10 to <55% (14). Serial evaluation of LVEF multiple gated acquisition scan (MUGA) is currently used widely to monitor for cardiotoxicity secondary to chemotherapeutic drugs. In comparison to two-dimensional echocardiography, MUGA has lower inter- and intra-observer variability in measurement of LVEF. However, it carries risk of radiation exposure and, like the two-dimensional (2D) echocardiogram, provides limited information regarding cardiac structure and diastolic function, which limits its ability to detect subclinical myocardial damage (15, 16).

**Figure 1:**
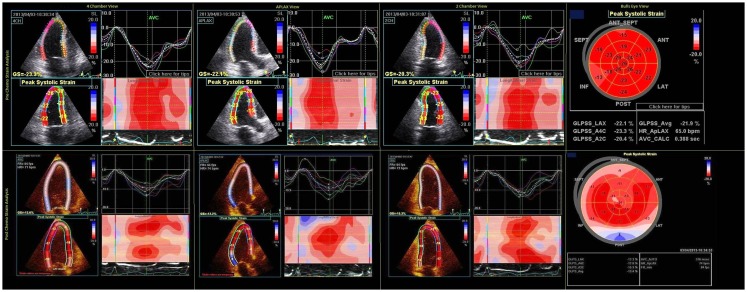
**Pre- and post-chemo strain imaging showing two chamber, three chamber, four chamber, and bull’s eye view**. The white and blue areas in ventricular strain imaging represent area of abnormal strain imaging. Global average longitudinal strain reduced from −21.9 to −13.4% after chemotherapy.

Three-dimensional (3D) echocardiography is reported to be more accurate than 2D echocardiogram in terms of intra- and inter-observer as well as test–retest variability (17) and cardiac magnetic resonance imaging for estimation of cardiac volumes and EF measurement (18). The myocardial motion during a systole is a complex phenomenon with shortening both longitudinally and circumferentially while thickening radially. Early cardiotoxic change in one of the myocardial motion can be compensated by another, giving a normal EF on testing. Chemotherapy-induced cardiotoxicity is regional, causing an ultrastructural damage that can precede the functional change of reduction in LVEF. Hence, assessing myocardial mechanics through deformation using strain analysis has emerged as a novel method to detect these early changes in myocardial function. Color tissue Doppler imaging uses the frequency shift between the original and tissue-reflected sonographic waves to calculate various cardiac functional parameters such as velocity, displacement, strain, and strain rate (SR). As the Doppler can only measure and detect changes in the direction of the sonographic beam, the Doppler-derived strain measurements have several restrictions such as angle dependency and inter-observer variability. Vector velocity imaging is another echocardiographic technique to quantitatively analyze myocardial mechanics, which is relatively angle independent. This technique is based on detecting frame-to-frame analysis of unique natural acoustic myocardial features referred to as “speckles.” These “speckles” from the myocardium in conjunction with 2D or 3D echocardiography are analyzed for motion in longitudinal, radial, and circumferential directions simultaneously. This is a semi-automated technique where manual delineation of the myocardium, followed by automated tracking software using a complex algorithm for the measurement of instantaneous velocity vector for individual speckles measured by analyzing their frame-to-frame spatial variability. These speckles are then added to give global values for myocardial functional parameters. The ideal tracking requires good image quality, optimum frame rate and manual readjustment of tracking if necessary for proper wall motion analysis by the software (Figure 1). The 3D analysis of the speckle tracking has the theoretic advantage of tracking the speckle in all of the three dimensions simultaneously, which is not possible with 2D speckle tracking and Doppler tracking, and therefore, permits comprehensive analysis of cardiac function. Unlike LVEF measurement, speckle tracking allows complex analysis of all the physiological myocardial activity during a cardiac cycle including movement in longitudinal, circumferential, and radial direction and measurement of the twist and torsion of the heart. In a few studies, the peak systolic radial, longitudinal, and circumferential strain decreases with elevation in plasma troponin have been validated to be early predictors of cardiotoxicity by anthracyclines and trastuzumab (19). In general, a reduction of longitudinal strain >10% from baseline after 3 months may predict future cardiac injury with a sensitivity and specificity of about 78 and 79%, respectively (19).

## Future Prospects for Prevention of Chemotherapy-Induced Cardiotoxicity

There is evidence of the use of angiotensin converting enzyme inhibitors (ACE-I) both for treatment and prophylaxis in chemotherapy-induced cardiotoxicity. Early treatment with these drugs seems to prevent and, to some extent, reverse the cardiotoxic effects of chemotherapeutic drugs (20). The US FDA has approved dexrazoxane, a derivative of ethylene-diamine tetraacetic acid (EDTA), for use in adults if cumulative doses of doxorubicin exceed 300 mg/m^2^ (4). It acts by preventing free radical formation at the cellular level but can also decrease the efficacy of some of chemotherapeutic agents by changing their pharmacokinetics. Other strategies are still under development and traditional approaches to reduce chemotherapy-induced cardiotoxicity including establishing stringent LVEF criteria for patient selection, monitoring cardiac function during therapy, and discontinuing potentially cardiotoxic therapy when cardiotoxicity arises still are the only ones available for clinicians currently.

## Integration of Specialties

Cardio-oncology and onco-cardiology are terms used to describe an integrated approach between cardiologists and oncologists. While chemotherapy is beneficial in destroying malignant cells, it can simultaneously cause injury or death to myocardial cells, which is described as cardiotoxicity. In the setting of neoadjuvant and adjuvant treatment and a laudable goal, a cancer survivor of today does not become the heart failure patient of tomorrow should be pursued.

Congestive heart failure contributes to the mortality and morbidity of cancer patients if not recognized early. In general, chemotherapeutic cardiac toxicity is classified as type 1 chemotherapy-related LV systolic dysfunction caused by agents such as doxorubicin, epirubicin, idarubicin, cyclophosphamide, and docetaxel. Type 2-mediated cardiotoxicity resulting from trastuzumab is generally not dose related and may be associated with reversible myocardial dysfunction. This class of agents also includes lapatinib, sunitinib, imatinib, and bevacizumab. This cardiac injury may occur early during the cancer treatment or may be delayed months to years after cancer has been successfully treated. Accurate cardiovascular monitoring at regular intervals during chemotherapy is particularly important with prolonged adjuvant therapy. With the use of vector velocity imaging or strain echocardiography, early detection of chemotherapy-induced cardiac injury is now within the realm of clinical practice. The aim of cardio-oncology collaboration is not to discontinue or reduce the dose of chemotherapy, which would reduce the efficacy of treatment, but to identify cardiotoxicity early and intervene so that congestive heart failure does not supervene.

The inter-disciplinary and integrative management of cancer patients with cardiovascular risks or patients who develop cardiovascular injury is: (a) early detection of patients at risk for cardiotoxicity; (b) early institution of cardioprotective agents; (c) preventing the mitigation of the chemotherapeutic agent as far as possible; (d) eliminating as much of the cancer as possible with the appropriate doses of chemotherapeutic agent while minimizing collateral damage, i.e., cardiotoxicity.

## Conclusion

Virtually all anti-cancer drugs target tumor cell death that may result in collateral injury to healthy tissues. Bone marrow suppression and gastrointestinal toxicities associated with chemotherapy are well recognized. Much less recognized, however, are the cardiotoxic effects of the cancer treatment. These side effects can cause systolic dysfunction, cardiac ischemia, cardiac arrhythmia, pericarditis, or chemotherapy-induced repolarization abnormalities. Common factors that increase a patient’s risk of developing cardiotoxic effects include cumulative dose, route of administration, age, prior irradiation, concomitant administration of other chemotherapeutics, and underlying heart disease. Radiation therapy (not discussed in this monograph) may result in coronary artery disease, valvular heart disease, pericardial injury, and myocardial disease from eventual fibrotic changes that occur post-radiation. Cardiovascular disease and cancer are the two leading causes of death in the USA; together they are responsible for nearly half of all deaths (21). As the survival population of the cancer patients increases, the acute and chronic cardiovascular effects of these drugs will become increasingly important. Therefore, the risk of cardiac toxicity should be balanced against the benefits of a particular chemotherapeutic agent based on individual case for optimal benefit to the patient. Much research is still needed to develop ideal guidelines to prevent or minimize cardiac injury in cancer patients undergoing chemotherapy. Early recognition using sensitive diagnostic techniques affords an opportunity for early treatment of these cardiotoxic effects. The Oncologist and cardiologist working in collaboration for patient care can ensure early diagnosis to improve quality of life and survival of the patients.

## Conflict of Interest Statement

The authors declare that the research was conducted in the absence of any commercial or financial relationships that could be construed as a potential conflict of interest.
